# An overview of the implementation of the first doctorate program in nursing in the Northern region

**DOI:** 10.1590/0034-7167.20257802301

**Published:** 2025-02-03

**Authors:** Janice de Matos Frazão, Mary Elizabeth de Santana

**Affiliations:** I Universidade do Estado do Pará. Belém, Pará, Brazil

The State University of Pará (UEPA) has just reached an important milestone in the academic and healthcare history of the Northern Region of Brazil with the implementation of the first academic doctorate program in nursing in the region, based in Belém^(1)^. This pioneering course represents a significant achievement and an essential response to the specific needs of the region, offering a new perspective and possibilities for training and research in the field of nursing.

The Northern Region is notoriously diverse and faces unique challenges in terms of public health. With its population spread over vast geographical areas and rich cultural diversity, the need for localized and specialized health solutions is evident^(2)^. The launch of this academic doctorate program is a strategic initiative to meet these demands, training professionals who not only understand the challenges of the region, but are also equipped to develop and implement innovative solutions.

The path to creating this course involved a careful analysis of regional needs and the development of a robust and adapted curriculum. The doctorate program in nursing at the State University of Pará is designed to integrate advanced theory and applied research, providing students with training that is both in-depth and practical. The focus on applied research will allow doctoral students to address local health issues with a critical and innovative eye, bringing direct benefits to the communities they serve.

A vital aspect of the program’s success was the selection of a highly qualified faculty. UEPA has brought together professionals with extensive experience and expertise in nursing and related fields, ensuring that the doctorate program not only meets the highest academic standards, but also offers relevant practical guidance. The presence of professors with experience in research and clinical practice is key to preparing doctoral students for complex challenges and promoting academic excellence.

The process of accrediting and validating the doctorate program was also a priority. Obtaining recognition from regulatory and accrediting agencies ensures that the course meets the highest standards of quality and credibility. This validation is fundamental to guaranteeing academic excellence and providing students with ethical training applied to the regional context.

The impact of this academic doctorate program in nursing will be vast and profound. The training of highly qualified professionals will not only contribute to improving health care in the Northern Region, but will also boost research and innovation in the area. This course will serve as a model of excellence, inspiring other institutions to follow UEPA’s example and expand the provision of advanced health education in the region.

Therefore, the implementation of the first academic doctorate program in nursing by the State University of Pará is a milestone for education and health care in the Northern Region. This advance not only fills a critical gap in academic training, but also reinforces UEPA’s commitment to excellence and innovation. The new offer represents the potential for a more promising future capable of tackling the region’s healthcare challenges, with benefits that will last for generations.
