# Supplementary material for: An overview of the implementation of the first doctorate program in nursing in the Northern region
Source: Rev Bras Enferm. 2025 Feb 3;78(3):e780301. doi: 10.1590/0034-7167.20257802301 (PMC11793647; doi:10.1590/0034-7167.20257802301)
Supplement: Translation

# Translation: O olhar sobre a implantação do primeiro doutorado em enfermagem da Região Norte

**DOI:** 10.1590/0034-7167.20257802301pt

**Authors:** Janice de Matos Frazão, Mary Elizabeth de Santana

**Affiliations:** I Universidade do Estado do Pará. Belém, Pará, Brasil

A Universidade do Estado do Pará (UEPA) acaba de marcar um importante capítulo na história acadêmica e na saúde da Região Norte do Brasil, com a implantação do primeiro curso de doutorado acadêmico em Enfermagem na região, sediado em Belém^(1)^. Esse curso pioneiro representa uma conquista significativa e uma resposta essencial às necessidades específicas da região, oferecendo uma nova perspectiva e novas possibilidades para a formação e pesquisa na área da Enfermagem.

A Região Norte é notoriamente diversa e enfrenta desafios únicos em termos de saúde pública. Com uma população espalhada por vastas áreas geográficas e uma rica diversidade cultural, a necessidade de soluções de saúde localizadas e especializadas é evidente^(2)^. O lançamento desse doutorado acadêmico é uma iniciativa estratégica para enfrentar essas demandas, formando profissionais que não só compreendem os desafios da região mas também estão equipados para desenvolver e implementar soluções inovadoras.

A trajetória para a criação desse curso envolveu uma cuidadosa análise das necessidades regionais e a elaboração de um currículo robusto e adaptado. O doutorado em Enfermagem da UEPA foi projetado para integrar teoria avançada e pesquisa aplicada, proporcionando aos alunos uma formação que é simultaneamente profunda e prática. O foco em pesquisa aplicada permitirá que os doutorandos abordem questões de saúde locais com um olhar crítico e inovador, trazendo benefícios diretos para as comunidades que atendem.

Um aspecto crucial para o sucesso do programa foi a formação de um corpo docente altamente qualificado. A UEPA reuniu profissionais com vasta experiência e expertise em Enfermagem e áreas relacionadas, garantindo que o doutorado não apenas atenda aos padrões acadêmicos mais elevados, mas também ofereça uma orientação prática relevante. A presença de professores com experiência em pesquisa e prática clínica é fundamental para preparar os doutorandos para desafios complexos e para promover a excelência acadêmica.

O processo de acreditação e validação do doutorado também foi uma prioridade. A obtenção do reconhecimento por órgãos reguladores e acreditadores assegura que o curso cumpra com os mais altos padrões de qualidade e credibilidade. Essa validação é fundamental para garantir a excelência acadêmica e para proporcionar aos alunos formação ética aplicada ao contexto regional.

O impacto desse doutorado acadêmico em Enfermagem será vasto e profundo. A formação de profissionais altamente qualificados não só contribuirá para a melhoria dos cuidados de saúde na Região Norte mas ainda impulsionará a pesquisa e a inovação na área. Esse curso servirá como um modelo de excelência, inspirando outras instituições a seguir o exemplo da UEPA e a expandir a oferta de educação avançada em saúde na região.

Portanto, a implantação do primeiro doutorado acadêmico em Enfermagem pela Universidade do Estado do Pará é um marco para a educação e a saúde na Região Norte. Esse avanço não só preenche uma lacuna crítica na formação acadêmica mas também reforça o compromisso da UEPA com a excelência e a inovação. A nova oferta representa uma promessa de um futuro mais promissor e capaz de enfrentar os desafios da saúde na região, com benefícios que se estenderão por gerações.

# Translation: Un vistazo a la implementación del primer doctorado en enfermería en la región Norte

**DOI:** 10.1590/0034-7167.20257802301es

**Authors:** Janice de Matos Frazão, Mary Elizabeth de Santana

**Affiliations:** I Universidade do Estado do Pará. Belém, Pará, Brasil

La Universidad del Estado de Pará (UEPA) acaba de escribir un importante capítulo en la historia académica y de salud en la Región Norte de Brasil con la implementación del primer doctorado académico en Enfermería de la región, localizado en Belém.^(1)^ Este curso pionero representa un importante logro y una respuesta fundamental a las necesidades específicas de la región, brindando una nueva perspectiva y posibilidades de capacitación e investigación en el campo de la Enfermería.

La Región Norte es notoriamente diversa y se enfrenta a desafíos únicos en materia de salud pública. Con una población distribuida en vastas áreas geográficas y una rica diversidad cultural, la necesidad de soluciones de salud localizadas y especializadas resulta evidente.^(2)^ El lanzamiento de este doctorado académico constituye una iniciativa estratégica para hacer frente a tales demandas, formando a profesionales que no solo comprendan los desafíos regionales, sino que estén capacitados para desarrollar e implementar soluciones innovadoras.

El itinerario para la creación de este curso incluyó un cuidadoso análisis de las necesidades regionales y la elaboración de un currículo robusto y adaptado. El doctorado en Enfermería de la UEPA ha sido concebido para integrar la teoría avanzada y la investigación aplicada, ofreciendo a los estudiantes una formación que sea a la vez profunda y práctica. El enfoque en la investigación aplicada permitirá a los estudiantes del doctorado abordar las cuestiones de salud locales con una visión crítica e innovadora, generando beneficios directos a las comunidades a las que atiendan.

Un aspecto clave para el éxito del programa ha sido la formación de un cuerpo docente altamente calificado. La UEPA ha reunido a profesionales con amplia experiencia y conocimientos en Enfermería y áreas relacionadas, garantizando que el doctorado no solo alcance los más altos estándares académicos, sino que también ofrezca una orientación práctica relevante. La presencia de profesores con experiencia en investigación y práctica clínica resulta fundamental para preparar a los estudiantes de doctorado para desafíos complejos y para promover la excelencia académica.

El proceso de acreditación y validación del doctorado también ha sido una prioridad. La obtención del reconocimiento de los organismos de regulación y acreditación asegura que el curso cumpla con los más altos estándares de calidad y credibilidad. Esta validación resulta fundamental para garantizar la excelencia académica y ofrecer a los estudiantes una formación ética aplicada al contexto regional.

El impacto de este doctorado académico en Enfermería será vasto y profundo. La formación de profesionales altamente calificados no solo contribuirá a mejorar la atención de salud en la Región Norte, sino que también impulsará la investigación y la innovación en la zona. Este curso servirá como modelo de excelencia, inspirando a otras instituciones a seguir el ejemplo de UEPA y a extender la oferta de educación avanzada en salud en la región.

Por ello, la implementación del primer doctorado académico en Enfermería en la Universidad del Estado de Pará constituye un hito para la educación y la salud en la Región Norte. Este avance no solo llena un vacío crítico en la formación académica, sino que refuerza el compromiso de la UEPA con la excelencia y la innovación. La nueva oferta representa la promesa de un futuro más prometedor, capaz de hacer frente a los desafíos de salud en la región, con beneficios que perdurarán por generaciones.
